# Health economic evaluation of structured education programs for patients with diabetes: a systematic review

**DOI:** 10.3389/fpubh.2024.1467178

**Published:** 2024-11-21

**Authors:** Caihua Ye, Qiwei Zhou, Wenfei Yang, Libo Tao, Xinjun Jiang

**Affiliations:** ^1^International Nursing School, Hainan Medical University, Haikou, Hainan, China; ^2^Center for Health Policy and Technology Evaluation, Peking University Health Science Center, Beijing, China

**Keywords:** health economic evaluation, diabetes, structured education, cost-effectiveness analysis, systematic review

## Abstract

**Background:**

Diabetes structured education programs have been demonstrated to effectively improve glycemic control and self-management behaviors. However, evidence on the health economic evaluation of these programs is limited.

**Objectives:**

To systematically review the health economic evaluation of structured education programs for patients with type 1 and type 2 diabetes mellitus.

**Methods:**

The English databases PUBMED, WEB OF SCIENCE, OVID, COCHRANE LIBRARY, EMBASE, and EBSCO, along with the Chinese databases CNKI, WANFANG, VIP, and SINOMED, were searched from their inception to September 2024. The quality of the literature was assessed using the CHEERS 2022 checklist. A descriptive analysis was performed on the studies included in the review, with all currencies converted to international dollars. An incremental cost-effectiveness ratio of less than one times the *per capita* GDP was considered highly cost-effective, while a ratio between one and three times the *per capita* GDP was considered cost-effective.

**Results:**

A total of 28 studies from upper-middle-income and high-income countries were included. The average quality score of the included studies was 18.6, indicating a moderate level of reporting quality. Among these, eleven studies demonstrated that diabetes structured education programs were highly cost-effective and twelve were found to be cost-effective. In contrast, three studies were deemed not cost-effective, and two studies provided uncertain results. The ranges of the incremental cost-effectiveness ratios for short-term, medium-term, and long-term studies were − 520.60 to 65,167.00 dollars, −24,952.22 to 14,465.00 dollars, and −874.00 to 236,991.67 dollars, respectively.

**Conclusion:**

This study confirms the cost-effectiveness of structured education programs for diabetes and highlights their importance for patients with type 2 diabetes who have HbA1c levels exceeding 7% and are receiving non-insulin therapy. Additionally, the potential advantages of incorporating telecommunication technologies into structured diabetes education were emphasized. These findings offer valuable insights and guidance for decision-making in diabetes management and clinical practice, contributing to the optimization of medical resource allocation and the improvement of health status and quality of life for patients.

## 1 Introduction

Diabetes mellitus is one of the most prevalent and severe chronic illnesses globally, posing a significant threat to public health worldwide (1). According to the International Diabetes Federation, the global prevalence of diabetes mellitus was 10.5% in 2021, amounting to approximately 536.6 million individuals, with the number of adult patients expected to rise to 783.2 million by 2045 (2). Diabetes mellitus can lead to various complications, including cardiovascular issues, kidney disease, eye problems, peripheral nerve damage, and stroke, among others (3). The chronic nature of diabetes and its associated complications places a substantial burden and significant economic strain on individuals, their families, and the entire healthcare system. A systematic review indicated that the average annual cost per person to treat type 2 diabetes ranged from 29.91 dollars to 237.38 dollars in low- and low middle-income countries (4). In 2021, global health expenditure on diabetes was projected to be 966 billion dollars, with adult diabetes expenditure estimated to reach 1,054 billion dollars by 2045 (2). A survey in the United States, found that nearly a quarter of individuals with diabetes reported spending an additional 300 dollars per month to manage their health (5). Consequently, identifying a cost-effective method for managing patients with diabetes is urgent.

Diabetes education is recognized as a fundamental aspect of diabetes care (6). Various effective educational interventions have been implemented to improve diabetes self-care (7). Structured education is one of the most essential forms of diabetes self-management education, and the International Consensus Guidelines on Diabetes highlight the need for structured diabetes self-management education programs for patients at the time of diagnosis (8). The Diabetes Structured Education Programs (DSEP) are defined as a self-management education that is systematic, structured, standardized, and personalized. These programs are developed and tailored by a professional diabetes education team, taking into account the patient’s education level, cultural background, health status, and individual needs (9, 10). Diabetes structured education programs have been implemented in numerous countries and have demonstrated significant clinical effects, such as improvements in HbA1c (11–13). Therefore, DSEP is prioritized and recommended to patients both international and multiple national diabetes guidelines (14–17).

Several studies have indicated that DSEP are cost-effective (18, 19). Jiang et al. evaluated the long-term cost-effectiveness of self-efficacy-centered structured diabetes education for non-insulin-treated type 2 diabetes patients from the perspective of the Chinese healthcare system (18). Using a 50-year simulation with the CORE model, the results indicated that structured education not only reduced patient complications but also increased life expectancy and quality-adjusted life years (QALYs), resulting in a savings of 5,221.97 dollars, with an incremental cost-effectiveness ratio (ICER) indicating dominance. However, the overall cost-effectiveness remains uncertain, particularly in health systems that lack sufficient resources to manage diabetes mellitus effectively (20). Wan et al. conducted a comprehensive search of five authoritative literature databases in Malaysia to systematically assess the cost-effectiveness of various educational interventions in diabetes management, including face-to-face education, structured education, and other models (21). Their analysis found that up to 89% of the literature supports the significant cost-effectiveness of these educational interventions. However, due to the variable overall quality of the included literature and the considerable methodological heterogeneity among the studies, it remains challenging to precisely identify the most cost-effective types of specific educational interventions. Therefore, the cost-effectiveness of structured education programs in diabetes management remains unclear. Additionally, two systematic reviews on diabetes self-management education programs suggested that such education is likely to be cost-effective (22, 23). Teljeur et al. conducted a systematic review in Ireland, which included 16 cost-analysis studies and 21 cost-effectiveness analyses. They concluded that the average cost of self-management education programs was 684 dollars, suggesting a likelihood of cost-effectiveness (23). However, both reviews acknowledged the heterogeneity of the included studies, making it difficult to determine which type of self-management education program was the most cost-effective. Furthermore, these reviews were published in 2017, and new evidence has emerged in recent years. Additionally, the focus of these reviews was not specifically on structured education programs. Therefore, it is essential to conduct a systematic review to analyze the cost-effectiveness of the DSEP.

In summary, both international and national diabetes guidelines emphasize the important of DSEP. While there have been original studies evaluating the economic effects of DSEP, as well as some reviews focusing on diabetes education, recent years have seen the emergence of new evidence. However, a systematic review specifically addressing the economic evaluation of structured education programs for patients with type 1 and type 2 diabetes is still absent. Conducting a systematic review on this topic is essential. Therefore, this study aims to systematically review the existing evidence of health economic evaluation studies concerning DSEP.

## 2 Methods

The Preferred Reporting Items for Systematic Reviews and Meta-Analyses (PRISMA Statement) guided the reporting of this systematic review (24).

### 2.1 Data resource and search strategy

Searches were conducted across six English databases (PUBMED, WEB OF SCIENCE, OVID, COCHRANE LIBRARY, EMBASE, and EBSCO) and four Chinese databases (CNKI, WANFANG, VIP, and SINOMED). The search strategy incorporated MeSH terms and entry words, tailored to each database. Additionally, manual searches and an inquiry into the ProQuest digital papers database were performed to obtain grey literature. The search terms included economic evaluation, cost effectiveness analysis (CEA), cost benefit analysis (CBA), cost utility analysis (CUA), cost minimization analysis (CMA), cost consequences analysis (CCA), diabetes mellitus, type 1 diabetes mellitus (T1DM), type 2 diabetes mellitus (T2DM), structured education (SE), structured education program (SEP), educational program, patient education, health education, health promotion, self-management, diabetes management program, diabetes education, and care program. The literature search period extended from the establishment of the database to September 2024.

### 2.2 Study selection

Two researchers independently selected the literature, resolving any disagreements through discussion or by involving a third researcher. Irrelevant literature was excluded by reviewing the title and abstract, with followed by a thorough reading of the full text to finalize inclusion based on the eligibility criteria. The PICOST (Population, Intervention, Comparison, Outcome, Study design, Time) framework guided the study selection process. Studies were included if they met the following criteria (i) population: patients with T1DM, T2DM, or both; (ii) interventions: any form of structured education program for individuals with diabetes; (iii) comparisons: any comparators such as usual care, standard care, or routine education; (iv) outcomes: cost, effectiveness indicators (e.g., HbA1c levels, complications, morbidity, mortality, life expectancy), cost-effectiveness indicators (e.g., ICER), cost-utility indicators (e.g., QALYs, disability-adjusted life years, health utility values, incremental cost-utility ratio (ICUR)), and cost–benefit indicators (e.g., net cost, incremental cost–benefit ratio); (v) study design: any research design related to health economic evaluation, including randomized controlled trials, prospective studies, retrospective studies, mixed-method designs, and modeling analyses; (vi) time: short- or long-term economic evaluations. Studies were excluded if they met any of the following criteria: (i) not published in Chinese or English; (ii) full text not available or if they were repeated publications; (iii) reviews, case reports, comments, protocols, and animal studies; (iv) incomplete content such as partial economic evaluations, those conducting only cost analysis, or lacking health outcome data; (v) patients who were pregnant or preparing for pregnancy; (vi) absence of a control group.

### 2.3 Data extraction

The retrieved data were imported into NoteExpress 3.9.0 software to eliminate duplicate literature. Two investigators independently screened, extracted, and analyzed the literature based on predefined inclusion and exclusion criteria. Any disagreements were resolved through discussion with a third researcher. A self-designed structured Excel form was utilized for data collection. For the selected studies, the extracted data included: (i) basic information (e.g., first author, title, publication year, country, income level), (ii) study characteristics (e.g., sample size, population, intervention, comparator, study design, data sources), and (iii) economic evaluation content (e.g., cost, perspective, time horizon, type of economic evaluation, discount rate, currency, health outcome, sensitivity analysis, quality score). If a study analyzed both short-term and long-term economic effects of DSEP, the long-term cost-effectiveness analysis was prioritized for reporting in this systematic review as it is more representative.

### 2.4 Quality assessment

The quality of the included studies was assessed using the 28-item checklist from the Consolidated Health Economic Evaluation Reporting Standards 2022 (CHEERS 2022) statement (25, 26). The CHEERS 2022 checklist is applicable for the standardized review of any type of health economic evaluation research reports, as well as for theoretical and empirical studies based on mathematical models. This checklist comprises seven main categories with 28 questions addressing various aspects of economic evaluations. Each question was scored as follows: 1 point for fully reported, 0.5 points for partially reported, and 0 points for not reported or not applicable (27). The total score, which ranges from 0 to 28, reflects the quality of study reporting, with higher scores indicating better quality. Quality assessments were conducted independently and cross-checked by two researchers, with any disagreements resolved through discussion with a third researcher.

### 2.5 Data analysis

To promote comparisons between studies, all currencies were converted into international dollars using the currency exchange rates provided by the World Bank (28). If a study did not report the year of the currency, the default was the publication year of the article. Additionally, according to the cost-effectiveness threshold method of *per capita* GDP recommended by WHO (29), ICER <1 times GDP *per capita*, with high cost effectiveness; 1 times *per capita* GDP < ICER <3 times *per capita* GDP, with cost effectiveness; ICER ≥3 times *per capita* GDP does not have effectiveness. Given the significant heterogeneity among the included studies, a descriptive analysis was employed to qualitatively evaluate the health economic evaluation studies.

## 3 Results

### 3.1 Study selection

**Figure 1:**
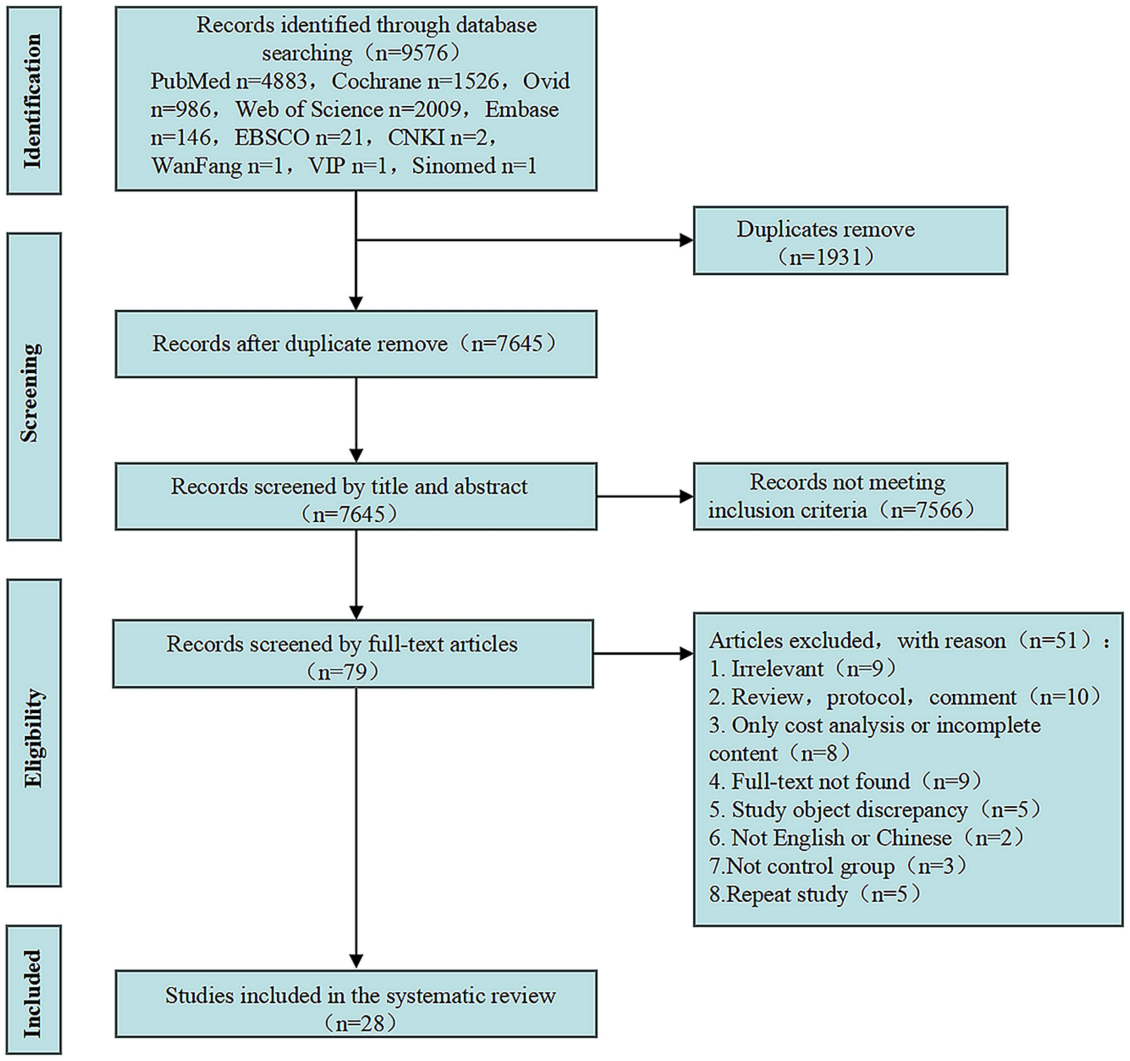
The PRISMA flow diagram for study selection.

A total of 9,576 relevant studies were retrieved from ten electronic literature databases. Following a review of the abstracts and full texts, 28 studies that met the inclusion criteria were selected. Specific details are presented in the PRISMA flow diagram (Figure 1).

### 3.2 Quality assessment

**Figure 2:**
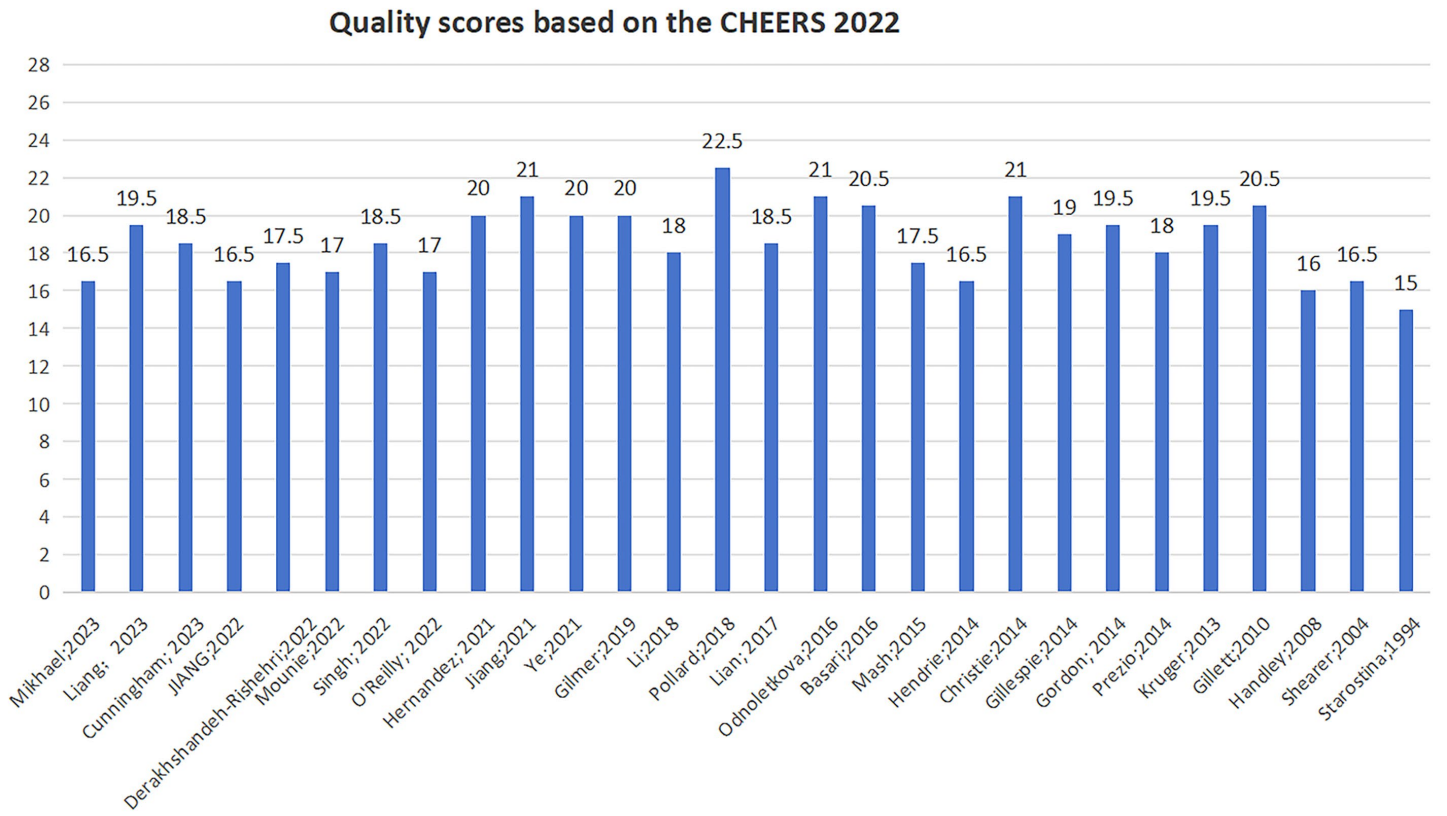
Quality scores based on the CHEERS 2022.

The quality assessment results of the included studies are presented in Figure 2. The scores for reporting quality ranged from 15.0 to 22.5, with a mean score of 18.6, indicating an overall moderate quality of reporting. The studies demonstrated stronger reporting in the title, abstract, background, results, and discussion sections, but weaker reporting in the research methods section. Regarding the health economic analysis plan (HEAP), only one study fully reported that the analysis was conducted according to a pre-developed plan (30). Four studies did not report the selected economic perspective (31–34), while 24 studies did report the perspective; however, most did not justify their choice. All included studies reported the research time horizon, but only one study provided the rationale for it (33). Concerning the research discount rate, only 16 studies reported this information (18, 19, 30, 31, 33, 35–45), and among these, 9 studies explained the reasons for their chosen discount rate (18, 19, 31, 33, 36, 38, 39, 42, 44). All studies reported the currency used, but 6 did not specify the currency reference year (31, 38, 44, 46–48). Among the 17 modeling studies, only (10) described whether the model was internally or externally validated (18, 30, 32, 33, 35, 37, 41–43, 45). Most studies mentioned their sources of funding; however, they did not adequately report the funding methods or the role of the funder in the study.

### 3.3 Research characteristics

The characteristics of the studies are summarized in Table 1. The 28 included studies, published between 1994 and 2023, comprised one study in Chinese (46) and 27 in English. These studies originated from 13 countries: Australia, Canada, Belgium, France, Ireland, Russia, the UK, the USA, China, Iran, Iraq, Mexico, and South Africa. Among them, 19 studies were from high-income countries (19, 30–41, 47–52) and 9 from upper-middle-income countries (18, 42–46, 53–55). Most studies employed a randomized controlled trial design, while others use cohort design (32, 42, 45), quasi-experimental design (33), and prospective controlled trial (40). Eighteen studies focused on participants with T2DM, seven on participants with T1DM, and three included participants with both types of diabetes. Sample sizes ranged from 78 to 40,548. The most commonly studied populations were non-insulin-treated patients (18, 32, 46, 55), those with moderately controlled diabetes (HbA1c > 7%) (34, 46, 47, 49, 55), newly diagnosed patients (< 1 year) (39, 55), and adults with T2DM. The content and delivery of the structured educational programs varied, with detailed descriptions of the DSEP provided in Table 1. The included studies primarily adopted perspectives from the healthcare systems, societal viewpoints, country-specific healthcare systems, payers, and patients. The types of costs considered varied and include direct medical costs, direct non-medical costs, and indirect costs. However, only three studies addressed both the direct and indirect costs associated with DSEP (40, 42, 44).

**Table 1:** Study characteristics.

First author, Year	Country	Study population; Sample size	Intervention	Comparator	Perspectives	Type of cost	Main outcome measure
Income level*: Upper middle income							
Mikhael, 2023 (54)	Iraq	Patients with diabetes; 78	The culturally specific diabetes self-management education and support programs	Usual care	The health care providers	Direct cost	ICER (HbA1c)
Liang, 2023 (42)	China	Patients with diabetes; 847	The integrated diabetes care program	Usual diabetes management	The societal perspective	Direct cost, indirect cost	ICUR (QALYs)
Jiang, 2022 (46)	China	HbA1c ≥ 7.5% in non-insulin-treated adults with T2DM; 265	The structured therapy and education programs	Usual care	The Chinese medical service system perspective	Direct cost	ICER (QALYs)
Derakhshandeh-Rishehri, 2022 (55)	Iran	Age > 20 years, diagnosed <1 years and HbA1c ≥ 7.0% non-insulin-dependent T2DM; 105	The weblog-telecommunication nutrition education program	Usual care	The patient perspective	Direct cost	ICER (HbA1c)
Jiang, 2021 (18)	China	Non-insulin therapy patients with T2DM; 265	The self-efficacy-focused structured education program	Routine education	The China’s healthcare service perspective	Direct cost	ICER (QALYs)
Hernandez, 2021 (45)	Mexico	Age 18–70 years and diagnosed <5 years patients with T2DM; 238	The multidisciplinary and comprehensive innovative diabetes self-management care program	Usual treatment	The payer’s perspective	Direct cost	ICER (QALYs)
Gilmer, 2019 (43)	Mexico	Patients with T2DM; 201	The technology-enhanced diabetes care management program	usual care	The health system perspective	Direct cost	ICER (QALYs)
Lian, 2017 (44)	China	Patients with T2DM; 23,162	The patient empowerment education program	usual care	The societal perspective	Direct cost, indirect cost	ICER (number of deaths avoided)
Mash, 2015 (53)	South Africa	Patients with T2DM; 1,570	The structured group education program	Usual care	The societal perspective	Direct cost	ICER (QALYs)
Income level*: high income							
Cunningham, 2023 (32)	UK	Non-insulin-treated patients with T2DM; 14,204	The self-management education of interactive website and mobile application	usual care	-	Direct cost	QALYs
Mounie, 2022 (49)	France	HbA1c 6.5–10% adult patients with T2DM; 256	The EDUC@DOM telemonitoring and tele-education program	Usual care	The payer perspective, the French National Health Insurance perspective	Direct cost	ICER (HbA1c)
Singh, 2022 (33)	UK	Patients with T2DM; 40,548	The WISDOM self-management education	Usual care	-	Direct cost	ICER (QALYs)
O’Reilly, 2022 (48)	Canada	Patients with T2DM; 365	The community-based, telephone-delivered diabetes health coaching intervention	Usual education	The public payer perspective	Direct cost	ICER (QALYs)
Ye, 2021 (37)	USA	Adult patients with diabetes; 222	The CHW + PL education program	The CHW-only education program	The healthcare sector perspective	Direct cost	ICER (QALYs)
Li, 2018 (50)	UK	Adult patients with T2DM; 374	The Web-based self-management education program	Usual care	The NHS and personal and social services perspective	Direct cost	ICER (QALYs)
Pollard, 2018 (30)	UK	Adult patients with T1DM; 267	The structured insulin pumps and DAFNE education	The structured MDI injections and DAFNE education	The NHS and personal and social services perspective	Direct cost	ICER (QALYs)
Odnoletkova, 2016 (35)	Belgium	18-75-year-old patients with T2DM on medication; 574	The nurse-led risk factor target-driven telephone self-management support program	Usual care	The perspective of the Belgian healthcare system	Direct cost	ICER (QALYs)
Basari (19)	UK	1-16-year-old patients with T1DM on multiple daily insulin injections; 480	The KICk-OFF structured education program	Usual care	The perspective of the UK National Health Service	Direct cost	ICER (QALYs)
Hendrie, 2014 (51)	Australia	Adult patients with T2DM; 245	The pharmacist-led Diabetes Management Education Program	Standard pharmacy care	The health sector’s perspective	Direct cost	ICER (the reduction in patients’ number of days with glycaemic episodes)
Christie, 2014 (36)	UK	Diagnosed ≥1 year and HbA1c ≥ 8.5% patients with T1DM; 362	The clinic-based structured educational group program	Routine care	The perspective of the NHS	Direct cost	ICER (QALYs)
Gillespie, 2014 (52)	Ireland	Patients with T1DM; 437	The group follow-up after participation in the DAFNE structured education program	Usual care	The perspective of the healthcare provider	Direct cost	QALYs
Prezio, 2014 (41)	USA	Patients with T2DM; 180	The one-to-one culturally tailored diabetes education and management program	Usual medical care	The health system perspective	Direct cost	ICER (QALYs)
Gordon, 2014 (34)	Australia	Aged 18–70 years, diagnosed >3 months and HbA1c ≥ 7.5% patients with T2DM; 120	The telephone-linked self-management education program	Usual care	-	Direct cost	ICER (QALYs)
Kruger, 2013 (38)	UK	Adult patients with T1DM; 5,000	The DAFNE structured education program	No training	The NHS perspective	Direct cost	ICER (QALYs)
Gillett, 2010 (39)	UK	Newly diagnosed patients with T2DM; 824	The diabetes education and self-management for ongoing and newly diagnosed program	Usual care	The NHS and personal social services perspective	Direct cost	Incremental cost (QALYs)
Handley, 2008 (47)	USA	Age > 17 years and HbA1c ≥ 8.0% patients with T2DM; 226	The automated telephone self-management support with nurse care management	Usual care	The health systems or program perspective	Direct cost	ICUR (QALYs)
Shearer, 2004 (31)	UK	Patients with T1DM; 100	The structured treatment and teaching program combining dietary freedom with insulin adjustment	Standard practice	-	Direct cost	Incremental life-years
Starostina, 1994 (40)	Russia	15-45-year-old insulin-dependent patients with T1DM; 181	The intensive treatment and teaching program based on urine glucose self-monitoring	The standard education program	The perspective of society as a whole	Direct cost, indirect cost	Net costs

*Income levels for different countries based on 2023 World Bank criteria; ICER, incremental cost-effectiveness ratio; ICUR, incremental cost-utility ratio; QALYs, quality-adjusted life years; CHW, community health worker; PL, peer leader; NHS, the National Health Service; DAFNE, the dose adjustment for normal eating; MDI, multiple daily insulin; KICk-OFF, kids in control of food; −, not reported or not applicable.

One study conducted a CBA (40), seven studies conducted CUA (30, 32, 39, 41, 42, 47, 48), and 20 studies conducted CEA. The included studies assessed DSEP over different durations: seven studies evaluated programs in the short-term (≤ 1 year), five studies in the medium-term (1–5 years), and sixteen studies over the long-term (> 5 years). Seventeen studies used decision analysis models to evaluate the economic outcomes of the DSEP over medium- and long-term periods. Among these, five studies used the Markov models (31, 34–36, 53), four used the United Kingdom Prospective Diabetes Study Outcome Model (32, 33, 42, 43), three used the Sheffield Type 1 Diabetes Policy models (19, 30, 38), two used the CORE Diabetes model (18, 45), one used the Sheffield Type 2 Diabetes Policy model (39), one used the Michigan model (37), one used the Archimedes model (41). The cost discount rates in the included studies ranged from 0 to 6%. Most articles reported the payment thresholds based on GDP *per capita*, willingness-to-pay, or purchasing power parity, with the exception of three articles that did not provide this information (31, 40, 47). Detailed health economic evaluations of the included studies are presented in Table 2.

**Table 2:** Detailed health economic evaluations of included studies.

First author, year	Study design	Economic evaluation; type of modeling	Time horizon	Currency; year of pricing; cost of discount rate	Threshold^*^	Costs per patient^*^	Outcome measures^*^	Sensitivity analysis	Result	Quality scores^#^
Short-term										
Mikhael, 2023 (54)	RCT	CEA; -	6 months	Iraqi Dinar; 2018/2019; -	1552.34–4828.66 dollars	Total cost: 20.90 dollars (intervention), 11.68 dollars (control); incremental cost: 9.22 dollars	An ICER of 15.61 dollars	Univariate sensitivity analysis	Highly cost-effective	16.5
Jiang, 2022 (46)	RCT	CEA; -	1 year	Yuan; 2017/2018; -	< 9039.39 dollars, 9039.39–27118.18 dollars	Total cost: 360.91 dollars (intervention), 418.91 dollars (control); incremental cost: −58 dollars	An ICER of −520.60 dollars	Multivariate sensitivity analysis	Highly cost-effective	16.5
Derakhshandeh-Rishehri, 2022 (55)	RCT	CEA; -	3 months	Dollar; 2021; -	< 13,116.00 dollars; 13,116–39,348.00 dollars	Total cost: 5,334.80 dollars (intervention), 634.16 dollars (control); incremental cost: 4,700.64 dollars	An ICER of 21,613.04 dollars	Univariate sensitivity analysis	Cost-effective	17.5
O’Reilly, 2022 (48)	RCT	CUA; -	1 year	Dollar; -; -	38,461.54 dollars	Total cost: 1216.15 dollars (intervention), 835.38 dollars (control); incremental cost: 380.77 dollars	An ICER of 27,022.31 dollars	Probabilistic sensitivity analysis	Cost-effective	17.0
Li, 2018 (50)	RCT	CEA; -	1 year	Pound; 2014; -	33,333.33–50,000.00 dollars	Total cost: 3465.00 dollars (intervention), 3445.00 dollars (control); incremental cost: 20.00 dollars	An ICER of 9,250.00 dollars	Univariate sensitivity analysis	Cost-effective	18.0
Hendrie, 2014 (51)	Block randomized	CEA; -	6 months	Dollar; 2011 -	< 39 dollars	Total cost: 356.00 dollars (intervention)	An ICER of 39.00 dollars	Scenario analysis	Cost-effective	16.5
Handley, 2008 (47)	RCT	CUA; -	1 year	Dollar; -; -	-	Total cost: 782.00 dollars (intervention)	An ICUR of 65,167.00 dollars	Univariate sensitivity analysis	Uncertain	16.0
Medium-term										
Mounie, 2022 (49)	RCT	CEA; -	2 years	Euro; 2020; -	WTP of threshold	Incremental cost: −5101.00 dollars	An ICER of −24,952.22 dollars	Probabilistic sensitivity analysis	Highly cost-effective	17.0
Lian, 2017 (44)	RCT	CEA; -	5 years	Dollar; -; 0%	Local estimate of the statistical value of life saved: < 1,282,051.28 dollars	Total cost: 247.00 dollars (intervention)	The ICER to avoid a death event was 14,465.00 dollars	Univariate sensitivity analysis	Cost-effective	18.5
Gillespie, 2014 (52)	RCT	CEA; -	18 months	Euro; 2009; -	< 7,142.86 dollars, 7,142.86–21,428.57 dollars	Total cost: 5,072.86 dollars (intervention), 6,195.71 dollars (control); incremental cost: −1122.85 dollars	The mean QALYs were 1.35 for control and 1.31 for intervention	Probability sensitivity analysis	Not cost-effective	19.0
Gordon, 2014 (34)	RCT + modeling	CEA; The Markov Model	5 years	Pound; 2011; -	< 55,000.00 dollars	Total cost: 28,586.67 dollars (intervention), 29,725.00 dollars (control); incremental cost: −1138.33 dollars	An ICER of dominant	Univariate sensitivity analyses, probabilistic sensitivity analysis, Scenario analyses	Highly cost-effective	19.5
Starostina, 1994 (40)	Prospective controlled trial	CBA; -	2 years	Rouble; 1992; 5%	-	Total cost: 17,666.67 dollars	Net costs within 2 years: −48,000.00 dollars	Univariate sensitivity analyses, multivariate sensitivity analysis	Uncertain	15.0
Long-term										
Cunningham, 2023 (32)	Cohort study + modeling	CUA; The UKPDS Outcomes Model	10 years	Pound; 2018; -	28,571.43 −42,857.14 dollars	Incremental cost: −169.60 dollars	An ICER of dominant	Univariate sensitivity analysis	Highly cost-effective	18.5
Liang, 2023 (42)	Cohort study + modeling	CUA; The UKPDS Outcomes Model	30 years	Yuan; 2021; 3%	< 12,652.50 dollars, 12,652.50 −37,957.50 dollars	Total cost: 15,625.00 dollars (intervention), 10,812.50 dollars (control); incremental cost: 4812.50 dollars	An ICUR of 16,042.19 dollars	-	Cost-effective	19.5
Singh, 2022 (33)	Quasi-experimental study + modeling	CEA; The UKPDS Outcome Model	lifetime	Pound; 2017/2018; 3.5%	< 14,285.71 dollars	Total cost: 47,004.29 dollars (intervention), 47,011.43 dollars (control); incremental cost: −7.14 dollars	An ICUR of 357.14 dollars	Probabilistic sensitivity analysis	Cost-effective	18.5
Hernandez, 2021 (45)	Cohort + modeling	CEA; The CORE Diabetes Model	20 years	Dollar; 2019; 5%	< 5000.00 dollars, 5000.00–10000.00 dollars	Total cost: 18138.00 dollars (intervention), 18819.00 dollars (control); incremental cost: −681.00 dollars	An ICER of −874.00 dollars	Probabilistic sensitivity analysis	Highly cost-effective	20.0
Jiang, 2021 (18)	RCT + modeling	CEA; The CORE Diabetes Model	50 years	Yuan; 2017/2018; 3.5%	< 8,993.94 dollars, 8,993.94–27,118.18 dollars	Incremental cost: −5,221.97 dollars	An ICER of dominant	Univariate sensitivity analysis, probabilistic sensitivity analysis	Highly cost-effective	21.0
Ye, 2021 (37)	RCT + modeling	CEA; The Michigan Model	20 years	Dollar; 2018; 3%	< 20,000.00 dollars, 20,000.00–100,000.00 dollars	Total cost: 128,435.00 dollars (intervention), 128,280.00 dollars (control); incremental cost: 155.00 dollars	An ICER of 5,900.00 dollars	Univariate sensitivity analysis, scenario analysis	Highly cost-effective	20.0
Gilmer, 2019 (43)	RCT + modeling	CEA; The UKPDS Outcomes Model	Lifetime	Dollar; 2017; 3%	< 9,064.00 dollars	Total cost: 45,442.00 dollars (intervention), 44,943.00 dollars (control); incremental cost: 499.00 dollars	An ICER of 2220.00 dollars	Probabilistic sensitivity analysis	Highly cost-effective	20.0
Pollard, 2018 (30)	RCT + modeling	CUA; The Sheffield Type 1 Diabetes Policy Model	Lifetime	Pound; 2013/2014; 3.5%	33,333.33–50,000.00 dollars	Total cost: 70,206.67 dollars (intervention), 33,048.33 dollars (control); incremental cost: 37,158.34 dollars	An ICER of 236,991.67 dollars	Probabilistic sensitivity analysis, scenario analysis	Not cost-effective	22.5
Odnoletkova, 2016 (35)	RCT + modeling	CEA; The Markov model	40 years	Euro; 2013; 3%	< 12,500.00 dollars	Incremental cost: 1,433.75 dollars	An ICER of 6,961.25 dollars	Univariate sensitivity analysis, scenario analysis	Highly cost-effective	21.0
Basari, 2016 (19)	RCT + modeling	CEA; The Sheffield Type 1 Diabetes Policy Model	Lifetime	Pound; 2011; 3.5%	< 33,333.33 dollars, 33,333.33–50,000.00 dollars	Total cost: 170,673.33 dollars (intervention), 168,798.33 dollars pounds (control); incremental cost: 1875.00 dollars	An ICER of 48,021.67 dollars	Probabilistic sensitivity analysis	Cost-effective	20.5
Mash, 2015 (53)	RCT + modeling	CEA; The Markov model	Lifetime	Dollar; -; -	< 6003.00 dollars, 6003.00–12006.00 dollars	Incremental cost: 125.00 dollars	An ICER of 1,862.00 dollars	Scenario analysis	Highly cost-effective	17.5
Christie, 2014 (36)	RCT + modeling	CEA; The Markov model	70 years	Pound; 2010/2011; 1.5%	33,333.33–50,000.00 dollars	Total cost: 413,288.33 dollars (intervention), 412,585.00 dollars (control); incremental cost: 703.33 dollars	An ICER of dominant	Univariate sensitivity analysis, probabilistic sensitivity analysis, scenario analysis	Not cost-effective	21.0
Prezio, 2014 (41)	RCT + modeling	CUA; The Archimedes Model	20 years	Dollar; 2012; 3%	< 50,000.00 dollars	Total cost: 4,958.00 dollars (intervention)	An ICER of 355.00 dollars	Scenario analysis; univariate sensitivity analysis	Cost-effective	18.0
Kruger, 2013 (38)	RCT + modeling	CEA; The Sheffield Type 1 Diabetes Policy Model	Lifetime	Pound; –; 3.5%	< 33,333.33 dollars	Total cost: 121,420.00 dollars (intervention), 120,710.00 dollars (control); incremental cost: 710.00 dollars	An ICER of 24,125.00 dollars	Probabilistic sensitivity analyses, structural sensitivity analyses	Cost-effective	19.5
Gillett, 2010 (39)	RCT + modeling	CUA; The Sheffield type 2 diabetes model	80 years	Pound; 2008; 3.5%	< 40,000.00 dollars	Total cost: 32,578.00 dollars (intervention), 32,160.00 dollars (control); incremental cost: 418.00 dollars	An ICER of 10,774.00 dollars	Univariate sensitivity analysis, probabilistic sensitivity analysis	Cost-effective	20.5
Shearer, 2004 (31)	RCT + modeling	CEA; The Markov model	10 years	Pound; -; 6%	-	Incremental cost: −4,474.00 dollars	Incremental life-years:0.05 years	Univariate sensitivity analysis, multivariate sensitivity analysis	Cost-effective	16.0

*All currencies were converted to international dollars; ^#^Quality assessment was undertaken using the CHEERS 2022 checklist; RCT, randomized controlled trial; CEA, cost-effectiveness analysis; ICER, incremental cost-effectiveness ratio; CUA, cost-utility analysis; ICUR, incremental cost-utility ratio; WTP, willingness to pay; QALYs, quality-adjusted life years; CBA, cost–benefit analysis; UKPDS, United Kingdom Prospective Diabetes Study; −, not reported or not applicable.

### 3.4 Effectiveness of the programs

In the CEA and CUA studies, the effectiveness indicators employed for health economic evaluation primarily included gained QALYs (18, 19, 30–39, 41–43, 45–48, 50, 52, 53), reduction in HbA1c (49, 54, 55), number of deaths avoided (44), increased life expectancy (18, 33, 35, 38, 43, 45), reduction in blood pressure (42, 54), weight reduction (54), improved lipid levels (54), enhanced fasting blood glucose (54), improvement in diabetes-related distress (50), and fewer days with glycemic episodes (51). Additionally, other studies considered effect indicators such as diabetes complications, hypoglycemia, hyperglycemic episodes, and BMI (19, 35, 44). Diabetes-related distress was assessed using the Problem Areas in Diabetes questionnaire (50). Furthermore, several studies evaluated the health utility values of patients through instruments such as the EuroQol 5 Dimensions scale (31, 35, 39, 46, 48, 50, 52), the Health Utilities Index Mark 2 (19), the Child Health Utility 9 Dimensions (19), the Short Form 36 functional status instrument (51), Short Form-12 Health Survey (47), and the visual analog scale (31). Another CBA study concentrated on calculating the costs and benefits of the DSEP (40).

### 3.5 Health economic evaluation

A total of 11 studies found that DSEP were highly cost-effective, while 12 studies indicated that they were cost-effective. In contrast, three studies reported that these programs were not cost-effective, and two studies yielded uncertain results. The outcomes of the health economic evaluations from these 28 studies are summarized in Table 2.

#### 3.5.1 Short-term

Seven studies evaluated the short-term cost-effectiveness of DSEP, covering periods ranging from of 3 months to 1 year (46–48, 50, 51, 54, 55). The results showed that the total cost of the interventions varied between 20.90 dollars and 5334.80 dollars, with incremental costs ranging from −58 dollars to 4,700.64 dollars. Notably, the structured therapy and education programs conducted in grass-roots areas of China by Jiang et al. (46) and the culturally specific diabetes programs in Iraq by Mikhael et al. (54) demonstrated that DSEP was highly cost effective, with ICER of −520.60 dollars and 15.61 dollars, respectively. Four other studies demonstrated cost-effectiveness, with ICER of 39 dollars, 9,250 dollars, 21,613.04 dollars, and 27,022.31 dollars (48, 50, 51, 55). In these cost-effectiveness studies, all participants were patients with T2DM, most of whom were adults with HbA1c level greater than 7% or non-insulin-treated type 2 diabetes (46, 50, 51, 55). In contrast, Handley et al. (47) evaluated the cost-utility of an automated telephone self-management support and nurse care management program in the USA over one year in patients with T2DM and an HbA1c ≥ 8.0%. This study found an increase of 0.012 QALYs in the intervention group compared to usual care, with a total intervention cost of 782 dollars and an ICUR of 65,167 dollars. However, while the study performed a univariate sensitivity analysis to address result uncertainty in the results, it did not consider cost-effectiveness thresholds for comparative analysis, leaving the cost-effectiveness of the project uncertain. Additionally, the studies by Jiang et al. (46), Derakhshandeh-Rishehri et al. (55), and Handley et al. (47) also assessed the QALYs gained from implementing DSEP, reporting increases of 0.042 years, 0.20 years, and 0.01 years, respectively.

#### 3.5.2 Medium-term

Five studies assessed the medium-term cost-effectiveness of DSEP, with durations ranging from 18 months to 5 years (34, 40, 44, 49, 52). The results indicated that the total cost of the interventions ranged from 247 dollars to 28,586.67 dollars, with incremental costs varying from −5,101 dollars to −1,122.85 dollars. Among these studies, the EDUC@DOM telemonitoring and tele-education program by Mounie et al. (49) and the telephone-linked self-management education program by Gordon et al. (34) both demonstrated that implementing DSEP for adults with T2DM and an average HbA1c greater than 7% was highly cost-effective, with ICER of 24,952.22 dollars and a dominating cost-effectiveness outcome, respectively. Lian et al. (44) found that DSEP was cost-effective in patients with T2DM, incurring a cost of 14,465 dollars to prevent one death event, while also reducing mortality, diabetes complications, and cardiovascular disease. Gillespie et al. (52) conducted a group follow-up after structured education program in Ireland, involving 437 patients with T1DM, and found that the structured education resulted in savings of 1,122.85 dollars. However, the intervention yielded a QALY of 1.31 years, which was 0.04 QALYs less than that of the control group, leading to the conclusion that this intervention was not cost-effective. Additionally, Starostina et al. (40) evaluated the cost–benefit of structured education programs in insulin-dependent patients with T1DM, finding improvements in metabolic control and cost savings in both groups. However, this study did not draw definitive conclusions regarding the cost-effectiveness outcomes, leaving the cost-effectiveness of the two groups uncertain.

#### 3.5.3 Long-term

Sixteen studies utilized health decision analysis models to evaluate the long-term cost-effectiveness of DSEP, with time horizons ranging from 10 years to a lifetime. The findings showed that the total cost of the interventions varied from 4,958 dollars to 413,288.33 dollars, while the incremental costs ranged from −5,221.97 dollars to 37,158.34 dollars. Among these studies, five reported that DSEP resulted in savings of approximately 7.14 dollars to 5,221.97 dollars (18, 31–33, 45); seven studies demonstrated that DSEP was highly cost-effective in patients with T2DM or diabetes, with ICER ranging from −874 dollars to 69,661.25 dollars, with some studies showing dominant cost-effectiveness (18, 32, 35, 37, 43, 45, 53); and seven studies indicated that DSEP was cost-effective, with ICER ranging from 355 dollars to 48,021.67 dollars (19, 31, 33, 38, 39, 41, 42). Most of the cost-effective studies focused on patients with T2DM, with Jiang et al. (18), Cunningham et al. (32), and Odnoletkova et al. (35) specifically examining non-insulin treated type 2 diabetes patients. Additionally, the incremental QALYs ranged from 0 to 0.34 years. However, two studies found no evidence of long-term cost-effectiveness. Pollard et al. conducted a lifetime cost-effectiveness analysis using the Sheffield Type 1 Diabetes Policy Model for structured insulin pumps and DAFNE education in patients with T1DM (30). This study reported an ICER of 236,991.67 dollars, exceeding the threshold of 33,333.33 dollars to 50,000.00 dollars, indicating that the intervention was not cost-effective and was associated with an increase in adverse events in the intervention group. Additionally, Christie et al. (36) used a Markov model to evaluate the 70-year cost-effectiveness of a clinic-based structured educational group program for patients with T1DM with HbA1c levels of ≥ 8.5%. The results showed that the ICER fell below the threshold of 38,767.66 dollars to 58,151.49 dollars; however, the intervention group did not showed improvements in metabolic control or gains in additional QALYs, and thus it was not considered cost-effective. Furthermore, Singh et al. (33), Kruger et al. (38), Jiang et al. (18), Gilmer et al. (43), Hernandez et al. (45), and Odnoletkova et al. (35) also projected life expectancy following the implementation of DSEP. The estimated increases in life expectancy were 0.01, 0.08, 0.2, 0.23, 0.5, and 1.18 years, respectively. The models were also used to simulate the occurrence of long-term complications, demonstrating that DSEP could reduce the incidence of diabetes-related complications, including myocardial infarction, stroke, atrial fibrillation, kidney failure, diabetic cardiovascular disease, diabetic kidney disease, heart failure, ischemic heart disease mortality, foot ulcers, foot amputations, diabetic neuropathy, and diabetic retinopathy (18, 31–33, 35, 38, 41, 45, 53).

### 3.6 Sensitivity analysis

Various sensitivity analyses were conducted in the included studies, encompassing univariate sensitivity analysis, multivariate sensitivity analysis, probabilistic sensitivity analysis (PSA), scenario analysis, and structural sensitivity analysis. The key indicators considered in these analyses mainly included glycated hemoglobin, discount rate, time horizon, cost of education programs, number of participants, health utility values and relevant thresholds. In the PSA, the probability that DSEP was cost-effective ranged from 14 to 100% (18, 19, 30, 33, 34, 36, 38, 39, 45, 48, 49, 52).

## 4 Discussion

This systematic review provides new evidence on the cost-effectiveness of structured education programs for individuals with T1DM and T2DM. By analyzing 28 studies from 13 countries, we found that DSEP are likely to be cost-effective in the short, medium and long term, leading to increased quality-adjusted life years, extended life expectancy, and a reduction in complications. These findings align with our initial hypothesis. Moreover, structured education programs for individuals with T2DM demonstrated better cost-effectiveness, particularly among patients on non-insulin therapy with HbA1c levels greater than 7%. However, the evidence for supporting the cost-effectiveness of structured education programs for individuals with T1DM remains limited, highlighting the need for further research to confirm these findings. These results offer valuable guidance for healthcare policymakers. Additionally, the combination of structured diabetes education with telecommunication technology shows promising potential for cost-effectiveness.

The results of this study indicate that, consistent with previous findings, structured education programs for diabetes can be cost-effective (21–23). Specifically, the most cost-effective interventions were the self-efficacy-focused structured education program (18), the multidisciplinary and comprehensive innovative diabetes self-management care program (45), the self-management education delivered via interactive websites and mobile applications (32), and the telephone-linked self-management education program (34). Several factors may contribute to these outcomes: structured education helps patients manage their condition more effectively, reducing medication costs and the risk of complications; the involvement of trained registered nurses at the grassroots level for education and follow-up lowers manpower costs; and the integration of telecommunication technology into structured education promotes long-term follow-up without the need for transportation, saving both time and money (35, 56, 57). However, the short-term cost-effectiveness of DSEP using telecommunication technology, as reported by Handley et al. (47), remains uncertain, and relevant studies are limited. Therefore, further evaluation of the long-term cost-effectiveness of telecommunication technology-based DSEP is necessary to support broader implementation. Additionally, our findings showed that DSEP is cost-effective for a subgroup of patients with type 2 diabetes who have HbA1c levels greater than 7% and are not treated with insulin. Some studies suggest that categorizing diabetes into subgroups based on demographic or disease-related factors can enable more personalized and targeted interventions, benefiting those most likely to see improvements, which has important implications for health policy and resource allocation (58, 59). This suggests that DSEP could serve as a precise and effective educational and management approach for this specific group. However, there is a limited amount of research using raw data in this area. Thus, further investigation is needed to explore the impact of structured education on the application and cost-effectiveness across different subgroups of T2DM.

While structured education programs for diabetes have showed positive outcomes, some studies suggest they may not always be cost-effective or yield conclusive results. These inconsistent findings are often linked to the inability of certain programs to improve blood sugar control, improve quality-adjusted life years, or manage high costs. In the structured educational cost-effectiveness study conducted by Pollard et al. (30), participants with T1DM treated with insulin pumps experienced high intervention costs, largely due to the expense of insulin pump therapy. In a 5-year follow-up study of patients with T1DM, Toresson et al. (60) assessed the costs associated with two different treatment modalities and found that insulin pumps were approximately 3,929 dollars more expensive per year than multiple daily insulin injections. This highlights the need for structured education programs to account for the cost differences between these two treatment modalities and consider the financial feasibility of the intervention for patients, families, and society. Additionally, studies by Starostina et al. (40) and Handley et al. (47) did not establish a cost-effectiveness threshold, which affected the evaluation of their results. Introducing such thresholds is crucial for determining cost-effectiveness and improving the scientific rigor of the assessments. Moreover, we observed that all studies reporting non-cost-effective outcomes involved patients with T1DM (30, 36, 52). This suggests that structured education programs for T1DM may not be cost-effective. However, given the limited evidence available, further investigation is necessary to draw more definitive conclusions.

In this systematic review, the majority of included studies adopted the perspective of the medical service system, with only four studies taking a broader societal perspective (40, 42, 44, 53). The economic burden of diabetes includes not only direct costs but also indirect and intangible costs (61–63). Due to the complexity and uncertainty in defining indirect and intangible costs, most studies focus exclusively on direct costs. It is recommended that future research adopt a societal perspective to evaluate the cost-effectiveness of programs, accounting for both direct and indirect costs, such as labor and productivity losses (e.g., absences from work or school) during illness. This approach would provide a more comprehensive view of the disease’s full economic burden and improve the accuracy of economic evaluations. Furthermore, among the 28 studies reviewed, the health economic studies of DSEP were primarily conducted in high- and middle-income countries, with a notable absence of evaluation from low-income countries. However, the economic burden of diabetes was particularly severe in low-income countries (64). A cross-sectional study showed that fewer than 10% of diabetes patients in low- and middle-income countries received comprehensive, guideline-based treatment (65), reflecting lower coverage of education programs compared to high-income countries. Additionally, several studies have demonstrated that DSEP in low-income countries can effectively control patient metabolism, reduce glycosylated hemoglobin levels, and improve self-management behaviors (66–68). Given the varying economic conditions across countries, future health economic evaluations of DSEP should focus on low-income countries to assess the program’s cost-effectiveness in these settings.

The majority of studies included in this review adhered to the CHEERS 2022 guidelines and achieved moderate levels of reporting quality, indicating that researchers recognize the importance of transparency and reproducibility. However, the quality of reporting in the research methods section remains insufficient. Precise documentation of research methods is essential for enabling other researchers to replicate or validate findings. Notably, only one study reported that its analysis was conducted according to a pre-established plan (30), suggesting that most studies may lack pre-registration or pre-planning, which could introduce bias into study design and execution. In the UK, it was found that only 30% of trials developed a HEAP as standard practice (69), despite health economists calling for HEAP standardization as early as 2008 (70). To improve the quality and transparency of health economics evaluation research, future studies should strengthen compliance with guidelines like CHEERS 2022, thereby improving the overall quality, reliability and applicability of research outcomes.

The findings of this study support the integration of structured educational programs into standard care practices for type 2 diabetes, particularly for patients with HbA1c levels greater than 7% who are not receiving insulin treatment. These results provide valuable guidance for health policymakers and researchers, helping them select the most appropriate and cost-effective DSEP and formulate more effective health policies aimed at improving health management and the quality of life for patients. Regarding future research directions: First, it is crucial to implement structured diabetes education using telecommunication technology in real-world settings to verify its clinical and cost-effectiveness and encourage wider adoption. Second, to deliver tailored educational programs for individuals with T2DM who stand to benefit most, it is important to investigate the effectiveness and cost-effectiveness of structured education across different subgroups, while also identifying their specific health needs and utilization patterns. Third, the cost-effectiveness of structured education for patients with T1DM appears suboptimal, highlighting the need for further research to develop a more cost-effective educational program. Lastly, multi-center studies that take social perspectives into account are recommended, particularly in low-income countries.

This study has several limitations. First, comparing interventions across studies is difficult due to the heterogeneity in the types and designs of DSEP, as well as differences in income levels and cultural backgrounds across countries. However, we have clearly defined DSEP in this context. Additionally, the World Health Organization (29) recommends that the cost-effectiveness of a program or intervention should be assessed based on the specific circumstances of each country or region, given varying levels of economic development. Therefore, we evaluated the cost-effectiveness of the programs strictly based on the threshold and ICER comparisons. Second, different studies adopt varying economic perspectives, mainly focusing on medical services and often overlooking indirect and intangible costs associated with structured education programs, which may impact the overall assessment of cost-effectiveness. Third, although we conducted a comprehensive search, we included only studies published in Chinese and English, which may have excluded relevant research in other languages. Finally, while we assessed the quality of the included studies, future evaluations should consider incorporating additional tools such as GRADE (71) and ECOBIAS (72, 73).

## 5 Conclusion

In conclusion, DSEP appears to be cost-effective in the long term for patients with T2DM, particularly those with HbA1c levels above 7% who are not receiving insulin therapy. The integration of telecommunication technology further improves both the effectiveness and cost-effectiveness of DSEP. However, the cost-effectiveness of DSEP for patients with T1DM requires further exploration. Given the variability in research quality and economic contexts, it is recommended to conduct multi-center, cross-border social perspective studies, especially in low-income countries, to comprehensively assess the cost-effectiveness of these programs. Such research will provide a scientific basis for policy development, optimize the allocation of medical resources, and ultimately improve the health and quality of life for patients.

## Data Availability

The original contributions presented in the study are included in the article/Supplementary material, further inquiries can be directed to the corresponding author.
