# Supplementary material for: Health economic evaluation of structured education programs for patients with diabetes: a systematic review
Source: Front Public Health. 2024 Nov 21;12:1467178. doi: 10.3389/fpubh.2024.1467178 (PMC11617538; doi:10.3389/fpubh.2024.1467178)
Supplement: Supplementary file 1 [file Table_1.docx]

***Supplementary Materials***

## 1 Search strategy

## 1.1 WEB OF SCIENCE: 2009 articles

((TS=(Economic evaluation OR cost effectiveness analysis OR cost benefit analysis OR cost utility analysis OR cost minimization analysis OR cost consequences analysis OR CEA OR CBA OR CUA OR CMA OR CCA)) AND TS=(Diabetes Mellitus OR Diabetes OR type 2 diabetes mellitus OR type 1 diabetes mellitus)) AND TS=(structured education OR structured education program OR educational program OR SE OR SEP patient education OR health education OR health promotion OR self-management OR diabetes management program OR diabetes education OR care program)

## 1.2 PUBMED: 4883 articles

((Economic evaluation OR cost effectiveness analysis OR cost benefit analysis OR cost utility analysis OR cost minimization analysis OR cost consequences analysis OR CEA OR CBA OR CUA OR CMA OR CCA) AND (("Diabetes Mellitus"[Mesh]) OR (Diabetes OR Diabetes Mellitus, Type 1 OR Diabetes Mellitus, Type 2 OR type 2 diabetes mellitus OR type 1 diabetes mellitus))) AND (structured education OR structured education programme OR educational programme OR SE OR SEP patient education OR health education OR health promotion OR self-management OR diabetes management program OR diabetes education OR care program)

## 1.3 OVID: 986 articles

(Economic evaluation OR cost effectiveness analysis OR cost benefit analysis OR cost utility analysis OR cost minimization analysis OR cost consequences analysis OR CEA OR CBA OR CUA OR CMA OR CCA) AND (Diabetes Mellitus OR Diabetes OR type 2 diabetes mellitus OR type 1 diabetes mellitus) AND (structured education OR structured education program OR educational program OR SE OR SEP patient education OR health education OR health promotion OR self-management OR diabetes management program OR diabetes education OR care program)

## 1.4 COCHRANE LIBRARY: 1526 articles

Economic evaluation OR cost effectiveness analysis OR cost benefit analysis OR cost utility analysis OR cost minimization analysis OR cost consequences analysis OR CEA OR CBA OR CUA OR CMA OR CCA in Title Abstract Keyword AND Diabetes Mellitus OR Diabetes OR type 2 diabetes mellitus OR type 1 diabetes mellitus in Title Abstract Keyword AND structured education OR structured education program OR educational program OR SE OR SEP OR patient education OR health education OR health promotion OR self-management OR diabetes management program OR diabetes education OR care program in Title Abstract Keyword - with Cochrane Library publication date Between Jan 1900 and Jan 2024

## 1.5 EBSCO: 21 articles

(Economic evaluation OR cost effectiveness analysis OR cost benefit analysis OR cost utility analysis OR cost minimization analysis OR cost consequences analysis OR CEA OR CBA OR CUA OR CMA OR CCA) AND (Diabetes Mellitus OR Diabetes OR type 2 diabetes mellitus OR type 1 diabetes mellitus) AND (structured education OR structured education program OR educational program OR SE OR SEP patient education OR health education OR health promotion OR self-management OR diabetes management program OR diabetes education OR care program)

## 1.6 EMBASE: 146 articles

('economic evaluation':ab,ti OR'cost effectiveness analysis':ab,ti OR'cost benefit analysis':ab,ti OR'cost utility analysis':ab,ti OR'cost minimization analysis':ab,ti OR'cost consequences analysis':ab,ti ORcea:ab,ti ORcba:ab,ti ORcua:ab,ti ORcma:ab,ti ORcca:ab,ti) AND ('diabetes mellitus':ab,ti ORdiabetes:ab,ti OR'type 2 diabetes mellitus':ab,ti OR'type 1 diabetes mellitus':ab,ti) AND ('structured education':ab,ti OR'structured education program':ab,ti OR'educational program':ab,ti ORse:ab,ti OR'sep patient education':ab,ti OR'health education':ab,ti OR'health promotion':ab,ti OR'self management':ab,ti OR'diabetes management program':ab,ti OR'diabetes education':ab,ti OR'care program':ab,ti)

## 1.7 CNKI: 2 articles

(卫生经济评价 OR 成本效果分析 OR 成本效益分析 OR 成本效用分析 OR 成本最小化分析) AND 糖尿病 AND 结构化教育

## 1.8 WANFANG: 1 article

(卫生经济评价 OR 成本效果分析 OR 成本效益分析 OR 成本效用分析 OR 成本最小化分析) AND 糖尿病 AND 结构化教育

## 1.9 VIP:1 article

(卫生经济评价 OR 成本效果分析 OR 成本效益分析 OR 成本效用分析 OR 成本最小化分析) AND 糖尿病 AND 结构化教育

## 1.10 Sinomed: 1 article

(卫生经济评价 OR 成本效果分析 OR 成本效益分析 OR 成本效用分析 OR 成本最小化分析) AND 糖尿病 AND 结构化教育

# 2 The CHEERS 2022 checklist and guidance for reporting.

| **Section/topic** | | **Item No** | **Guidance for reporting** |
| --- | --- | --- | --- |
| **Title** | Title | 1 | Identify the study as an economic evaluation and specify the interventions being compared. |
| **Abstract** | Abstract | 2 | Provide a structured summary that highlights context, key methods, results, and alternative analyses. |
| **Introduction** | Background and objectives | 3 | Give the context for the study, the study question, and its practical relevance for decision making in policy or practice. |
| \| **Methods** \| \| --- \| \|  \| \|  \| \|  \| \|  \| \|  \| \|  \| \|  \| \|  \| \|  \| \|  \| \|  \| \|  \| \|  \| \|  \| \|  \| \|  \| \|  \| | Health economic analysis plan | 4 | Indicate whether a health economic analysis plan was developed and where available. |
|  | Study population | 5 | Describe characteristics of the study population (such as age range, demographics, socioeconomic, or clinical characteristics). |
|  | Setting and location | 6 | Provide relevant contextual information that may influence findings. |
|  | Comparators | 7 | Describe the interventions or strategies being compared and why chosen. |
|  | Perspective | 8 | State the perspective(s) adopted by the study and why chosen. |
|  | Time horizon | 9 | State the time horizon for the study and why appropriate. |
|  | Discount rate | 10 | Report the discount rate(s) and reason chosen. |
|  | Selection of outcomes | 11 | Describe what outcomes were used as the measure(s) of benefit(s) and harm(s). |
|  | Measurement of outcomes | 12 | Describe how outcomes used to capture benefit(s) and harm(s) were measured. |
|  | Valuation of outcomes | 13 | Describe the population and methods used to measure and value outcomes. |
|  | Measurement and valuation of resources and costs | 14 | Describe how costs were valued. |
|  | Currency, price date, and conversion | 15 | Report the dates of the estimated resource quantities and unit costs, plus the currency and year of conversion. |
|  | Rationale and description of model | 16 | If modelling is used, describe in detail and why used. Report if the model is publicly available and where it can be accessed. |
|  | Analytics and assumptions | 17 | Describe any methods for analyzing or statistically transforming data,  any extrapolation methods, and approaches for validating any model used. |
|  | Characterizing heterogeneity | 18 | Describe any methods used for estimating how the results of the study vary for subgroups. |
|  | Characterizing distributional effects | 19 | Describe how impacts are distributed across different individuals or adjustments made to reflect priority populations. |
|  | Characterizing uncertainty | 20 | Describe methods to characterize any sources of uncertainty in the analysis. |
|  | Approach to engagement with patients and others affected by the study | 21 | Describe any approaches to engage patients or service recipients, the general public, communities, or stakeholders (such as clinicians or payers) in the design of the study |
| \| **Results** \| \| --- \| \|  \| \|  \| \|  \| | Study parameters | 22 | Report all analytic inputs (such as values, ranges, references) including uncertainty or distributional assumptions. |
|  | Summary of main results | 23 | Report the mean values for the main categories of costs and outcomes of interest and summarize  them in the most appropriate overall measure. |
|  | Effect of uncertainty | 24 | Describe how uncertainty about analytic judgments, inputs, or projections affects findings. Report the effect of choice of discount rate and time horizon, if applicable. |
|  | Effect of engagement with patients and others affected by the study | 25 | Report on any difference patient/service recipient, general public, community,  or stakeholder involvement made to the approach or findings of the study |
| **Discussion** | Study findings, limitations, generalizability, and current knowledge | 26 | Report key findings, limitations, ethical or equity considerations not captured,  and how these could affect patients, policy, or practice. |
| \| **Other relevant information** \| \| --- \| \|  \| | Source of funding | 27 | Describe how the study was funded and any role of the funder in the identification, design, conduct, and reporting of the analysis |
|  | Conflicts of interest | 28 | Report authors conflicts of interest according to journal or International Committee of Medical Journal Editors requirements. |

# 3 Quality assessment of included studies based on the CHEERS 2022 checklist.

| **First author; years** | **Quality scores** |
| --- | --- |
| Mikhael (54); 2023 | 16.5 |
| JIANG (49); 2022 | 16.5 |
| Derakhshandeh-Rishehri (55); 2022 | 17.5 |
| O'Reilly (48); 2022 | 17 |
| Li (51); 2018 | 18 |
| Hendrie (52); 2014 | 16.5 |
| Handley (47); 2008 | 16 |
| Mounie (50); 2022 | 17 |
| Lian (44); 2017 | 18.5 |
| Gillespie (53); 2014 | 19 |
| Gordon (34); 2014 | 19.5 |
| Starostina (40); 1994 | 15 |
| Cunningham (32); 2023 | 18.5 |
| Liang (42); 2023 | 19.5 |
| Singh (33); 2022 | 18.5 |
| Hernandez (45); 2021 | 20 |
| Jiang (57); 2021 | 21 |
| Ye (37); 2021 | 20 |
| Gilmer (43); 2019 | 20 |
| Pollard (30); 2018 | 22.5 |
| Odnoletkova (35); 2016 | 21 |
| Basari (19); 2016 | 20.5 |
| Mash (46); 2015 | 17.5 |
| Christie (36); 2014 | 21 |
| Prezio (41); 2014 | 18 |
| Kruger (38); 2013 | 19.5 |
| Gillett (39); 2010 | 20.5 |
| Shearer (31); 2004 | 16 |

# 4.Tables

## 4.1 Table 1

**Table 1. Study characteristics**

| **First author;**  **Year** | **Country** | **Study population; Sample size** | **Intervention** | **Comparator** | **Perspectives** | **Type of cost** | **Main outcome measure** |
| --- | --- | --- | --- | --- | --- | --- | --- |
| **Income level*****: Upper middle income** | | | | | | | |
| Mikhael (54); 2023 | Iraq | Patients with diabetes; 78 | The culturally specific diabetes self-management education and support programs | Usual care | The health care providers | Direct cost | ICER (HbA1c) |
| Liang (42); 2023 | China | Patients with diabetes;  847 | The integrated diabetes care program | Usual diabetes management | The societal perspective | Direct cost, indirect cost | ICUR(QALYs) |
| JIANG (49); 2022 | China | HbA1c ≥ 7.5% in non-insulin-treated adults with T2DM; 265 | The structured therapy and education programs | Usual care | The Chinese medical service system perspective | Direct cost | ICER (QALYs) |
| Derakhshandeh-Rishehri (55); 2022 | Iran | Age >20 years, diagnosed < 1 years and HbA1c ≥ 7.0% non-insulin-dependent T2DM; 105 | The weblog-telecommunication nutrition education program | Usual care | The patient perspective | Direct cost | ICER (HbA1c) |
| Jiang (18); 2021 | China | Non-insulin therapy patients with T2DM; 265 | The self-efficacy-focused structured education program | Routine education | The China's healthcare service perspective | Direct cost | ICER (QALYs) |
| Hernandez (45); 2021 | Mexico | Age 18-70 years and diagnosed < 5 years patients with T2DM;  238 | The multidisciplinary and comprehensive innovative diabetes self-management care program | Usual treatment | The payer’s perspective | Direct cost | ICER (QALYs) |
| Gilmer (43); 2019 | Mexico | Patients with T2DM;  201 | The technology-enhanced diabetes care management program | usual care | The health system perspective | Direct cost | ICER (QALYs) |
| Lian (44); 2017 | China | Patients with T2DM;  23,162 | The patient empowerment education program | usual care | The societal perspective | Direct cost, indirect cost | ICER (number of deaths avoided) |
| Mash (46); 2015 | South Africa | Patients with T2DM; 1,570 | The structured group education program | Usual care | The societal perspective | Direct cost | ICER (QALYs) |
| **Income level*****: High income** | | | | | | | |
| Cunningham (32); 2023 | UK | Non-insulin-treated patients with T2DM;  14,204 | The self-management education of interactive website and mobile application | usual care | - | Direct cost | QALYs |
| Mounie (50); 2022 | France | HbA1c 6.5-10% adult patients with T2DM; 256 | The EDUC@DOM telemonitoring and tele-education program | Usual care | The payer perspective; the French National Health Insurance perspective | Direct cost | ICER (HbA1c) |
| Singh (33); 2022 | UK | Patients with T2DM;  40,548 | The WISDOM self-management education | Usual care | - | Direct cost | ICER (QALYs) |
| O'Reilly (48); 2022 | Canada | Patients with T2DM;  365 | The community-based, telephone-delivered diabetes health coaching intervention | Usual education | The public payer perspective | Direct cost | ICER (QALYs) |
| Ye (37); 2021 | USA | Adult patients with diabetes; 222 | The CHW + PL education program | The CHW-only education program | The healthcare sector perspective | Direct cost | ICER (QALYs) |
| Li (51); 2018 | UK | Adult patients with T2DM; 374 | The Web-based self-management education program | Usual care | The NHS and personal and social services perspective | Direct cost | ICER (QALYs) |
| Pollard (30); 2018 | UK | Adult patients with T1DM; 267 | The structured insulin pumps and DAFNE education | The structured MDI injections and DAFNE education | The NHS and personal and social services perspective | Direct cost | ICER (QALYs) |
| Odnoletkova (35); 2016 | Belgium | 18-75-year-old patients with T2DM on medication; 574 | The nurse-led risk factor target-driven telephone self-management support program | Usual care | The perspective of the Belgian healthcare system | Direct cost | ICER (QALYs) |
| Basari (19); 2016 | UK | 1-16-year-old patients with T1DM on multiple daily insulin injections; 480 | The KICk-OFF structured education program | Usual care | The perspective of the UK National Health Service | Direct cost | ICER (QALYs) |
| Hendrie (52); 2014 | Australia | Adult patients with T2DM; 245 | The pharmacist-led Diabetes Management Education Program | Standard pharmacy care | The health sector's perspective | Direct cost | ICER (the reduction in patients’ number of days with glycaemic episodes) |
| Christie (36); 2014 | UK | Diagnosed ≥ 1 year and HbA1c ≥ 8.5% patients with T1DM; 362 | The clinic-based structured educational group program | Routine care | The perspective of the NHS | Direct cost | ICER (QALYs) |
| Gillespie (53); 2014 | Ireland | Patients with T1DM;  437 | The group follow-up after participation in the DAFNE structured education program | Usual care | The perspective of the healthcare provider | Direct cost | QALYs |
| Prezio (41); 2014 | USA | Patients with T2DM; 180 | The one-to-one culturally tailored diabetes education and management program | Usual medical care | The health system perspective | Direct cost | ICER (QALYs) |
| Gordon (34); 2014 | Australia | Aged 18-70 years, diagnosed >3 months and HbA1c ≥ 7.5% patients with T2DM; 120 | The telephone-linked self-management education program | Usual care | - | Direct cost | ICER (QALYs) |
| Kruger (38); 2013 | UK | Adult patients with T1DM; 5,000 | The DAFNE structured education program | No training | The NHS perspective | Direct cost | ICER (QALYs) |
| Gillett (39); 2010 | UK | Newly diagnosed patients with T2DM; 824 | The diabetes education and self-management for ongoing and newly diagnosed program | Usual care | The NHS and personal social services perspective | Direct cost | Incremental cost (QALYs) |
| Handley (47); 2008 | USA | Age >17 years and HbA1c ≥ 8.0% patients with T2DM;  226 | The automated telephone self-management support with nurse care management | Usual care | The health systems or program perspective | Direct cost | ICUR(QALYs) |
| Shearer (31); 2004 | UK | Patients with T1DM; 100 | The structured treatment and teaching program combining dietary freedom with insulin adjustment | Standard practice | - | Direct cost | Incremental life-years |
| Starostina (40); 1994 | Russia | 15-45-year-old insulin-dependent patients with T1DM; 181 | The intensive treatment and teaching program based on urine glucose self-monitoring | The standard education program | The perspective of society as a whole | Direct cost, indirect cost | Net costs |

*: Income levels for different countries based on 2023 World Bank criteria; ICER: incremental cost-effectiveness ratio; ICUR: incremental cost-utility ratio; QALYs: quality-adjusted life years; CHW: community health worker; PL: peer leader; NHS: the National Health Service; DAFNE: the dose adjustment for normal eating; MDI: multiple daily insulin; KICk-OFF: kids in control of food; -: not reported or not applicable.

## 4.2 Table 2

**Table 2. Detailed health economic evaluations of included studies.**

| **First author; year** | **Study design** | **Economic evaluation; type of modeling** | **Time horizon** | **Currency; year of pricing; cost of discount rate** | **Threshold^*^** | **Costs per patient^*^** | **Outcome measures^*^** | **Sensitivity analysis** | **Result** | **Quality scores^#^** |
| --- | --- | --- | --- | --- | --- | --- | --- | --- | --- | --- |
| **Short periods:** | | | | | | | | | | |
| Mikhael (54); 2023 | RCT | CEA; - | 6 months | Iraqi Dinar; 2018/2019; - | 1552.34 - 4828.66 dollars | Total cost: 20.90 dollars (intervention), 11.68 dollars (control); incremental cost: 9.22 dollars | An ICER of 15.61 dollars | Univariate sensitivity analysis | Highly cost-effective | 16.5 |
| JIANG (49); 2022 | RCT | CEA; - | 1 year | Yuan; 2017/2018; - | < 9039.39 dollars, 9039.39 - 27118.18 dollars | Total cost: 360.91 dollars (intervention), 418.91 dollars (control); incremental cost: -58 dollars | An ICER of -520.60 dollars | Multivariate sensitivity analysis | Highly cost-effective | 16.5 |
| Derakhshandeh-Rishehri (55); 2022 | RCT | CEA; - | 3 months | Dollar; 2021; - | < 13,116.00 dollars; 13,116 - 39,348.00 dollars | Total cost: 5,334.80 dollars (intervention), 634.16 dollars (control); incremental cost: 4,700.64 dollars | An ICER of 21,613.04 dollars | Univariate sensitivity analysis | Cost-effective | 17.5 |
| O'Reilly (48); 2022 | RCT | CUA;  - | 1 year | Dollar;  -  - | 38,461.54 dollars | Total cost: 1216.15 dollars (intervention), 835.38 dollars (control); incremental cost: 380.77 dollars | An ICER of 27,022.31 dollars | Probabilistic sensitivity analysis | Cost-effective | 17.0 |
| Li (51); 2018 | RCT | CEA; - | 1 year | Pound; 2014; - | 33,333.33 - 50,000.00 dollars | Total cost: 3465.00 dollars  (intervention), 3445.00 dollars  (control); incremental cost: 20.00 dollars | An ICER of 9,250.00 dollars | Univariate sensitivity analysis | Cost-effective | 18.0 |
| Hendrie (52); 2014 | Block randomized | CEA; - | 6 months | Dollar; 2011 - | < 39 dollars | Total cost: 356.00 dollars (intervention) | An ICER of 39.00 dollars | Scenario analysis | Cost-effective | 16.5 |
| Handley (47); 2008 | RCT | CUA;  - | 1 year | Dollar;  -  - | - | Total cost: 782.00 dollars (intervention) | An ICUR of 65,167.00 dollars | Univariate sensitivity analysis | Uncertain | 16.0 |
| **Medium periods:** | | | | | | | | | | |
| Mounie (50); 2022 | RCT | CEA; - | 2 years | Euro; 2020; - | WTP of threshold | Incremental cost: -5101.00 dollars | An ICER of -24,952.22 dollars | Probabilistic sensitivity analysis | Highly cost-effective | 17.0 |
| Lian (44); 2017 | RCT | CEA;  - | 5 years | Dollar;  -;  0% | Local estimate of the statistical value of life saved: < 1,282,051.28 dollars | Total cost: 247.00 dollars (intervention) | The ICER to avoid a death event was 14,465.00 dollars | Univariate sensitivity analysis | Cost-effective | 18.5 |
| Gillespie (53); 2014 | RCT | CEA; - | 18 months | Euro; 2009; - | < 7,142.86 dollars, 7,142.86 - 21,428.57 dollars | Total cost: 5,072.86 dollars (intervention), 6,195.71 dollars (control); incremental cost: - 1122.85 dollars | The mean QALYs were 1.35 for control and 1.31 for intervention | Probability sensitivity analysis | Not cost-effective | 19.0 |
| Gordon (34); 2014 | RCT + modeling | CEA;  The Markov Model | 5 years | Pound;  2011;  - | < 55,000.00 dollars | Total cost: 28,586.67 dollars (intervention), 29,725.00 dollars (control); incremental cost: - 1138.33 dollars | An ICER of dominant | Univariate sensitivity analyses, probabilistic sensitivity analysis, Scenario analyses | Highly cost-effective | 19.5 |
| Starostina (40); 1994 | Prospective controlled trial | CBA; - | 2 years | Rouble; 1992; 5% | - | Total cost: 17,666.67 dollars | Net costs within 2 years: - 48,000.00 dollars | Univariate sensitivity analyses, multivariate sensitivity analysis | Uncertain | 15.0 |
| **Long periods:** | | | | | | | | | | |
| Cunningham (32); 2023 | Cohort study + modeling | CUA;  The UKPDS Outcomes Model | 10 years | Pound;  2018;  - | 28,571.43 - 42,857.14 dollars | Incremental cost: -169.60 dollars | An ICER of dominant | Univariate sensitivity analysis | Highly cost-effective | 18.5 |
| Liang (42); 2023 | Cohort study + modeling | CUA;  The UKPDS Outcomes Model | 30 years | Yuan;  2021;  3% | < 12,652.50 dollars, 12,652.50 - 37,957.50 dollars | Total cost: 15,625.00 dollars (intervention), 10,812.50 dollars (control); incremental cost: 4812.50 dollars | An ICUR of 16,042.19 dollars | - | Cost-effective | 19.5 |
| Singh (33); 2022 | Quasi-experimental study + modeling | CEA;  The UKPDS Outcome Model | lifetime | Pound;  2017/2018;  3.5% | < 14,285.71 dollars | Total cost: 47,004.29 dollars (intervention), 47,011.43 dollars (control); incremental cost: - 7.14 dollars | An ICUR of 357.14 dollars | Probabilistic sensitivity analysis | Cost-effective | 18.5 |
| Hernandez (45); 2021 | Cohort + modeling | CEA;  The CORE Diabetes Model | 20 years | Dollar;  2019;  5% | < 5000.00 dollars, 5000.00 - 10000.00 dollars | Total cost: 18138.00 dollars (intervention), 18819.00 dollars (control); incremental cost: - 681.00 dollars | An ICER of -874.00 dollars | Probabilistic sensitivity analysis | Highly cost-effective | 20.0 |
| Jiang (18); 2021 | RCT + modeling | CEA; The CORE Diabetes Model | 50 years | Yuan; 2017/2018; 3.5% | < 8,993.94 dollars, 8,993.94 - 27,118.18 dollars | Incremental cost: -5,221.97 dollars | An ICER of dominant | Univariate sensitivity analysis,  probabilistic sensitivity analysis | Highly cost-effective | 21.0 |
| Ye (37); 2021 | RCT + modeling | CEA; The Michigan Model | 20 years | Dollar; 2018; 3% | < 20,000.00 dollars, 20,000.00 - 100,000.00 dollars | Total cost: 128,435.00 dollars (intervention), 128,280.00 dollars (control); incremental cost: 155.00 dollars | An ICER of 5,900.00 dollars | Univariate sensitivity analysis, scenario analysis | Highly cost-effective | 20.0 |
| Gilmer (43); 2019 | RCT + modeling | CEA;  The UKPDS Outcomes Model | Lifetime | Dollar;  2017;  3% | < 9,064.00 dollars | Total cost: 45,442.00 dollars (intervention), 44,943.00 dollars (control); incremental cost: 499.00 dollars | An ICER of 2220.00 dollars | Probabilistic sensitivity analysis | Highly cost-effective | 20.0 |
| Pollard (30); 2018 | RCT + modeling | CUA; The Sheffield Type 1 Diabetes Policy Model | Lifetime | Pound; 2013/2014; 3.5% | 33,333.33 - 50,000.00 dollars | Total cost: 70,206.67 dollars (intervention), 33,048.33 dollars (control); incremental cost: 37,158.34 dollars | An ICER of 236,991.67 dollars | Probabilistic sensitivity analysis, scenario analysis | Not cost-effective | 22.5 |
| Odnoletkova (35); 2016 | RCT + modeling | CEA; The Markov model | 40 years | Euro; 2013; 3% | < 12,500.00 dollars | Incremental cost: 1,433.75 dollars | An ICER of 6,961.25 dollars | Univariate sensitivity analysis, scenario analysis | Highly cost-effective | 21.0 |
| Basari (19); 2016 | RCT + modeling | CEA; The Sheffield Type 1 Diabetes Policy Model | Lifetime | Pound; 2011; 3.5% | < 33,333.33 dollars, 33,333.33 - 50,000.00 dollars | Total cost: 170,673.33 dollars (intervention), 168,798.33 dollars pounds (control); incremental cost: 1875.00 dollars | An ICER of 48,021.67 dollars | Probabilistic sensitivity analysis | Cost-effective | 20.5 |
| Mash (46); 2015 | RCT + modeling | CEA; The Markov model | Lifetime | Dollar; - - | < 6003.00 dollars, 6003.00 – 12006.00 dollars | Incremental cost: 125.00 dollars | An ICER of 1,862.00 dollars | Scenario analysis | Highly cost-effective | 17.5 |
| Christie (36); 2014 | RCT + modeling | CEA; The Markov model | 70 years | Pound; 2010/2011 1.5% | 33,333.33 - 50,000.00 dollars | Total cost: 413,288.33 dollars (intervention), 412,585.00 dollars (control); incremental cost: 703.33 dollars | An ICER of dominant | Univariate sensitivity analysis, probabilistic sensitivity analysis, scenario analysis | Not cost-effective | 21.0 |
| Prezio (41); 2014 | RCT + modeling | CUA; The Archimedes Model | 20 years | Dollar; 2012 3% | < 50,000.00 dollars | Total cost: 4,958.00 dollars (intervention) | An ICER of 355.00 dollars | Scenario analysis; univariate sensitivity analysis | Cost-effective | 18.0 |
| Kruger (38); 2013 | RCT + modeling | CEA; The Sheffield Type 1 Diabetes Policy Model | Lifetime | Pound; - 3.5% | < 33,333.33 dollars | Total cost: 121,420.00 dollars (intervention), 120,710.00 dollars (control); incremental cost: 710.00 dollars | An ICER of 24,125.00 dollars | Probabilistic sensitivity analyses, structural sensitivity analyses | Cost-effective | 19.5 |
| Gillett (39); 2010 | RCT + modeling | CUA; The Sheffield type 2 diabetes model | 80 years | Pound; 2008; 3.5% | < 40,000.00 dollars | Total cost: 32,578.00 dollars (intervention), 32,160.00 dollars (control); incremental cost: 418.00 dollars | An ICER of 10,774.00 dollars | Univariate sensitivity analysis, probabilistic sensitivity analysis | Cost-effective | 20.5 |
| Shearer (31); 2004 | RCT + modeling | CEA; The Markov model | 10 years | Pound; - 6% | - | Incremental cost: - 4,474.00 dollars | Incremental life-years:0.05 years | Univariate sensitivity analysis, multivariate sensitivity analysis | Cost-effective | 16.0 |

*: All currencies were converted to international dollars; #: Quality assessment was undertaken using the CHEERS 2022 checklist; RCT: randomized controlled trial; CEA: cost-effectiveness analysis; ICER: incremental cost-effectiveness ratio; CUA: cost-utility analysis; ICUR: incremental cost-utility ratio; WTP: willingness to pay; QALYs: quality-adjusted life years; CBA: cost-benefit analysis; UKPDS: United Kingdom Prospective Diabetes Study; -: not reported or not applicable.
